# Outcomes of superior capsule reconstruction for massive rotator cuff tears and risk factors for postoperative retear

**DOI:** 10.1007/s00402-019-03316-2

**Published:** 2019-12-05

**Authors:** Satoru Ohta, Osamu Komai, Yuuki Onochi

**Affiliations:** Department of Orthopaedic Surgery, Shinseikai Toyama Hospital, 89-10, Shimowaka, Imizu-shi, Toyama, 939-0243 Japan

**Keywords:** Superior capsule reconstruction, Massive rotator cuff tear, Postoperative retear, Surgical indications of SCR

## Abstract

**Introduction:**

Superior capsule reconstruction (SCR) has been used for the tendon grafting of massive rotator cuff tears when primary repair is difficult. We examined the postoperative outcomes of SCR for massive rotator cuff tears and the risk factors for postoperative retear.

**Materials and methods:**

Through this retrospective comparative study, we evaluated 35 patients with an average age of 75.3 (57–90) years who underwent SCR using the technique developed by Mihata et al. Clinical outcomes were evaluated 1 year postoperatively using the Japan Orthopedic Association (JOA) score, University of California Los Angeles (UCLA) shoulder score, elevation angle and the Sugaya classification, which uses a 5-point scale evaluation on magnetic resonance imaging in which types 4 and 5 are considered retears. We also investigated the progression of fatty degeneration before and after surgery and the rupture site of the graft. Acromio-humeral distance (AHD), before and after surgery was measured through X-rays. Rotator cuff tear-related shoulder arthritis was evaluated on plain X-rays using the Hamada stage. Risks of retear were identified using multiple regression analyses for sex, age, Hamada stage and JOA score.

**Results:**

The JOA score improved from 62.3 ± 9.49 (SD) preoperatively to 84.6 ± 5.66 (SD) postoperatively (*P* < 0.001). The UCLA score improved from 15.3 ± 3.77 (SD) preoperatively to 30.1 ± 3.11 (SD) postoperatively (*P* < 0.001). AHD increased from 4.03 mm preoperatively to 6.23 mm postoperatively (*P* < 0.001).

Postoperative retear was observed in seven of the 35 patients. Moreover, retear was observed in five of nine patients with a Hamada stage ≥ 4. Multiple regression analysis revealed that age ≥ 80 years, male sex and Hamada stage ≥ 4 were risk factors for retear.

**Conclusions:**

While the postoperative outcomes of SCR are favorable, age, sex and degree of arthropathic change should be considered for surgical indications of SCR.

## Introduction

Until now, many studies have reported on the procedures used for massive rotator cuff tear, e.g., debridement [1], biceps tenotomy [2, 3], partial repair [4, 5], patch graft [6–8], latissimus dorsi transfer [9, 10] and reverse total shoulder arthropathy (RSA) [11, 12]. However, it is not easy to obtain satisfactory results via any of the surgical methods and symptoms, such as dysfunction and pain, may persist after surgery.

As a treatment strategy for symptomatic massive rotator cuff tears, in 2010, Bedi et al. [13] graded recommendation levels of each surgical method based on the levels of evidence; surgical procedures employing tissue augmentation were classified as grade C. Although ≤ stage 2 of the Goutallier classification [14] is considered an indicator of primary repair, since 2009, we have been performing arthroscopic patch graft surgeries using the tensor fascia lata for large-to-massive rotator cuff tears with advanced fatty degeneration of < stage 3. However, after using this method, retears were observed in almost all cases with severe degeneration of the supraspinatus and infraspinatus tendons resulting in the tendon stump being pulled inside the glenoid fossa [15, 16]. Therefore, in 2011, we adapted the superior capsule reconstruction (SCR) method developed by Mihata et al. [17–20]. Although SCR has been recently developed in Japan and has only a short history, it reportedly has better advantages than arthroscopic patch graft surgeries in terms of retear rate, elevation angles and indications [15, 16]. Further, SCR may be a rational treatment option for relatively young patients with joint-conserving surgery and non-recoverable RCT. In contrast, it is a technically difficult procedure that requires extension of operation time and the risk of associated complications, such as infection, often increases; moreover, the materials needed and the effects of using SCR are not yet established [21]. Satisfactory results have been reported in SCR which surpasses other treatment outcomes and is becoming popular, and few studies have reported on the postoperative results and indications. Here we assessed (1) the outcome after SCR and determined (2) the risk factors for retear after SCR.

## Materials and methods

### Patient selection

This retrospective study included 35 patients who underwent SCR and was approved by the ethics committee of Shinseikai Toyama Hospital, Japan. In addition, informed consent was obtained from all patients included in this study. All surgeries for massive rotator cuff tears for which primary repair was difficult were performed between 2011 and 2015 by the same surgeon. Few reports have mentioned the condition of primary repair. Arthroscopy during surgery was used to determine if primary repair was possible. The primary repair was considered difficult if the torn tendon did not reach the original footprint even after sufficient mobilization [20] and when it arrived as primary repair. In addition, torn tendon reaches to the inside of greater tuberosity; cases where partial repair was possible were not indicated for this operation. The exclusion criteria were anterior upward migration such as Hamada classification type V, cervical spondylotic muscular atrophy, axillary nerve palsy, deltoid dysfunction and infection. There were 2 of 35 cases in which only SCR was performed without immobilization because there was no instability for massive rotator cuff tears with a history of shoulder joint dislocation. This study also included patients with grade 3 or higher fatty degeneration of the supraspinatus and infraspinatus tendons, according to the Goutallier classification [14] on preoperative magnetic resonance imaging (MRI). Plain X-rays of the shoulders of patients with cuff tear-related arthritis were evaluated using the Hamada stage score [22]; stage 1 (*n* = 9) was indicated by minimal radiographic changes, stage 2 (*n* = 15) by narrowing of the subacromial space to < 5 mm, stage 3 (*n* = 3) by erosion and acetabulization of the acromion caused by superior migration of the humeral head, stage 4a (*n* = 4) by glenohumeral arthritis and stage 4b (*n* = 5) by acetabulization and stage 5 (*n* = 0) by the presence of humeral head osteonecrosis. SCR was not medically indicated toward stage 5 cases; hence, no such patient was included in the study.

### Patient assessment

The patients were periodically assessed from the preoperative to the final postoperative observation stages. Patient assessments were conducted by three of our hospital's senior doctors and specialists. Shoulder joint range of motion (ROM), including flexion and internal and external rotation without shoulder abduction, was measured at every visit and the Japan Orthopedic Association (JOA) and University of California Los Angeles (UCLA) scores were used for clinical evaluation. In addition, the occurrence of complications during and after surgery was assessed.

### Surgical technique

Surgery was performed using the traction method with the patient in the lateral decubitus position (two-directional) under general anesthesia. The rotator cuff tear size was measured after confirming the difficulty to pull the tendons to greater tuberosity with sufficient attempts to mobilize the cuff tendon under arthroscopy. We measured the length (mediolateral dimension) and width (anteroposterior dimension) using a probe and created a 3–5-cm large tear and 5 cm or more massive tear. The fascia of the disinfected ipsilateral thigh was collected, and a graft of 6–8 mm thickness was prepared. In patients with a history of ipsilateral total hip arthroplasty, the iliotibial band was collected for graft preparation. The medial side of the graft was sutured to the glenoid superior tubercle and the lateral side was sutured using the suture-bridge method after inserting an anchor lateral into the greater tuberosity, in accordance with the procedure reported by Mihata et al. [20]. The posterior end of the graft was sutured to the infraspinatus tendon in a side-to-side fashion.

### Postoperative rehabilitation

Postoperative rehabilitation was performed in accordance with the rehabilitation program for large-to-massive cuff tears followed at our hospital. The shoulder joint was stabilized using a shoulder abduction orthosis for 3 weeks postoperatively. Hand and elbow movements were resumed on postoperative day 2. Passive shoulder ROM training (elevation in the scapular plane) was initiated from postoperative week 3, internal and external rotation from week 4 and the abduction brace was removed during postoperative week 6. Active-assisted exercise was initiated from postoperative week 6 or 7, whereas active movements and resistive exercise were allowed from postoperative week 8 or 9 and from postoperative week 12 onward.

### X-ray evaluation

Before surgery, 1 year after surgery, and at final observation, we acquired radiography images in four directions: front and rear directions in the middle of the upper arm, oblique frontal view, axial view and scapular Y-view. We also evaluated AHD. Radiographic assessments were conducted by three of our hospital's senior doctors and specialists.

### Assessment of repair integrity

The state of postoperative rotator cuff repair was evaluated using MRI performed with a 1.5-T scanner (Siemens Symphony; Siemens, Munich, Germany). MRI was performed at 3, 6, and 12 months after surgery and every year thereafter, and the results were double checked with a radiologist. Repair integrity was classified on the sagittal and coronal sections of T2-weighted images, according to a five-point scale of Sugaya classification [23]; type I was indicated by sufficient thickness with homogeneously low intensity, type II by sufficient thickness with partial high intensity, type III by insufficient thickness without discontinuity, type IV by the presence of a minor discontinuity and type V by the presence of a major discontinuity. Types 4 and 5 indicated either retear or non-healing. For evaluation of cuff integrity of graft tendon using Sugaya classification, MRI evaluation was performed before and after surgery in all patients. We performed MRI at 3 months, 6 months, 1 year and 2 years after surgery. For 18 patients, we could not perform MRI during the second year. Furthermore, according to the classification of Lim et al. [24, 25] as a graft tear pattern, type 1 graft tear was defined as a failure at lateral row of the anchor in which no tissue remained on the footprint and type 2 graft tear was defined as a failure that occurred immediately medial to the medial-row of the anchor in which remnant graft tissue was observed at the insertion site. In addition, the progression of fatty degeneration in SSP, ISP and SubS was investigated using Goutallier classification [14].

### Statistical analysis

Statistical data were analyzed using the *χ*^2^ test, Wilcoxon signed-rank test and multiple regression analyses (with sex, age, Hamada stage and JOA scores as the adjusted factors). *P* < 0.05 was considered statistically significant.

## Results

The study included 35 patients [18 males and 17 females; mean age: 75.3 (SD 7.17; range 57–90) years] who were followed up for at least 1 year postoperatively [mean follow-up duration: 40.6 (SD 17.1; range 12–62) months]. As a graft, the iliotibial band was obtained from four patients and the tensor fascia lata was obtained from 31 patients (Table 1). The pre- and post-operative JOA and UCLA scores improved from 62.3 (SD 9.49; range 40–78) to 84.6 (SD 5.66; range 74–98) (*P* < 0.001) and from 15.3 (SD 3.77; range 8–23) to 30.1 (SD 3.11; range 23–35) points (*p* < 0.001), respectively.

**Table 1:** Patient characteristics

Characteristic	Categories	Number (percent)
Age at surgery (years)	Mean	75.3
	SD	± 7.17
Sex	M	18 (51.4)
	F	17 (48.6)
Graft	Tensor fascialata	31 (88.6)
	Iliotibial band	4 (11.4)
Hamada classification	Stage 1	9 (25.7)
	Stage 2	15 (42.9)
	Stage 3	2 (5.7)
	Stage 4a	4 (11.4)
	Stage 4b	5 (14.3)
	Stage 5	0 (0)

*M* male, *F* female

AHD improved from 4.03 (SD 1.71; range 1.6–8.2) mm preoperatively to 6.23 (SD 1.72; range 2.8–10.1) mm (*P* < 0.001) at 1 year postoperatively.

In the no-retear group, it improved from 3.63 (SD 1.44; range 1.6–7.8) mm preoperatively to 6.62 (SD 1.46; range 2.8–10.1) mm (*p* < 0.001) postoperatively, but no change was observed in the retear group from 5.24 (SD 2.12; range 2.7–8.2) mm preoperatively to 4.39 (SD 0.96; range 3.3–6) mm (*P* = 0.575) postoperatively (Table 2).

**Table 2:** Results of AHD and fatty degeneration

	Preoperative	Postoperative	*P* value
AHD (mm)			
Total	4.03 ± 1.71	6.23 ± 1.72	< 0.001
No-retear group	3.63 ± 1.44	6.62 ± 1.46	< 0.001
Retear group	5.24 ± 2.12	4.39 ± 0.96	0.575
Fatty degeneration			
SSP	3.09 ± 0.78	3.31 ± 0.76	0.069
ISP	2.00 ± 0.80	2.09 ± 0.85	0.529
SubS	1.57 ± 0.70	1.49 ± 0.70	0.109

*AHD* acromio-humeral distance, *SSP* supraspinatus, *ISP* infraspinatus, *SubS* subscapularis

**Fig. 1:**
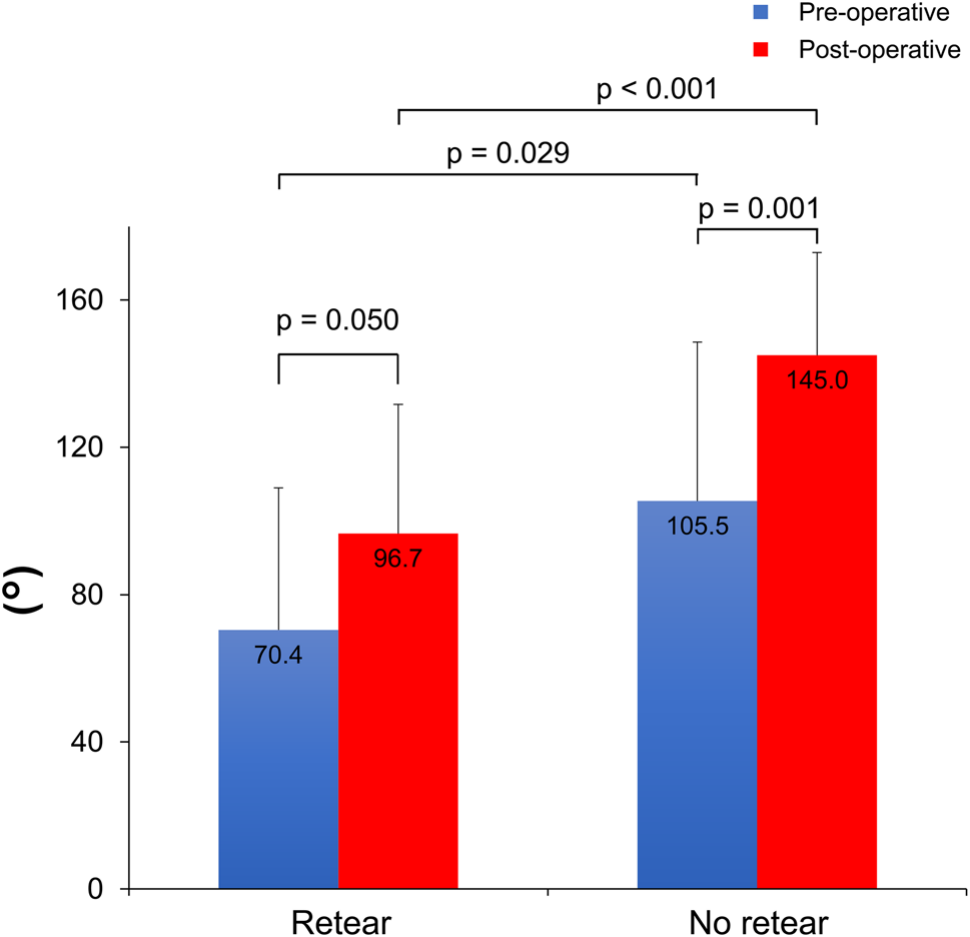
Elevation angles after surgery. Significant differences were observed in the elevation angle after surgery between the retear and no-retear groups

In seven patients (20%; six males; five were aged ≥ 80 years), retear occurred within 1 year postoperatively. In all seven patients, retear was confirmed by MRI at 3 months. Among the 7 cases, the retear at the graft site was of type 1 in 5 (71.4%) and type 2 in 2 (28.6%). When comparing pre- and post-operative JOA scores according to the presence or absence of retear, significant improvement was observed; the pre- and post-operative scores were 59.9 (SD 9.44; range 46–72) and 81.4 (SD 4.47; range 74–88) (*P* = 0.018) in the retear group and 62.9 (SD 9.58; range 40–78) and 85.4 (SD 5.71; range 74–98) (*P* < 0.001) in the no-retear group, respectively. Regarding the scapular elevation angle, no significant difference was observed in the retear group pre- and post-operatively [70.4° (SD 56.7°; range 20°–160°) vs. 96.7° (SD 53.7; range 30°–170°), respectively; *P* = 0.050]; however, in the no-retear group, a significant difference was observed [105.5° (SD 53.6°; range 0°–170°) vs. 145.0° (SD 38.5°; range 60°–180°), respectively; *P* = 0.001]. Furthermore, a significant difference was noted in the post-operative elevation angle (*P* < 0.001; Fig. 1) between the retear and no-retear groups.

Furthermore, the retear rate was 7.4% and 62.5% for patients with Hamada stages 1–3 and stage ≥ 4, respectively (*P* = 0.003). Multiple regression analyses revealed that the risk factors for retear included age ≥ 80 years (*P* = 0.014), Hamada stage ≥ 4 (*P* = 0.034) and male sex (*P* = 0.040) (Table 3). Both preoperatively and postoperatively, neither SSP, ISP nor SubS showed improvement or progress of fatty degeneration (Table 2). In the retear and no-retear groups, preoperative fatty degeneration was 3.13 (SD 0.99) and 3.07 (SD 0.73) (*P* = 0.834) for SSP, 2.10 (SD 1.20) and 2.00 (SD 0.60) (*P* = 0.809) for ISP and SubS of 1.75 (SD 1.04) and 1.52 (SD 0.58) (*P* = 0.773) (Table 4). Regarding complications, anchor dislodgement at the greater tuberosity was observed in one patient during surgery and severe inflammatory synovitis, which was treated by arthroscopic debridement, was observed postoperatively in one. None of the patients had postoperative infections at the graft surgical or harvest sites.

**Table 3:** Results of the multiple regression analyses

	Co-efficient	Standard error	*P* value
Age ≥ 80 years	0.349	0.133	0.014
Male sex	0.237	0.111	0.040
Preoperative JOA	− 0.008	0.006	0.188
Postoperative JOA	− 0.002	0.011	0.880
Hamada stage 4	0.296	0.133	0.034

The multiple regression analyses revealed that age ≥ 80 years (*P* = 0.014), Hamada stage ≥ 4 (*P* = 0.034) and male sex (*P* = 0.040) were risk factors for postoperative retear

Coefficient of determination representing the degree of influence; the closer the value is to 1, the higher is the impact

*JOA* Japan Orthopedic Association score

**Table 4:** Comparison of preoperative fatty degeneration between the retear and no-retear groups

Preoperative	Retear group	No-retear group	*P* value
SSP	3.13 ± 0.99	3.07 ± 0.73	0.834
ISP	2.10 ± 1.20	2.00 ± 0.60	0.809
SubS	1.75 ± 1.04	1.52 ± 0.58	0.773

*SSP* supraspinatus, *ISP* infraspinatus, *SubS* subscapularis

## A case of retear

**Fig. 2:**
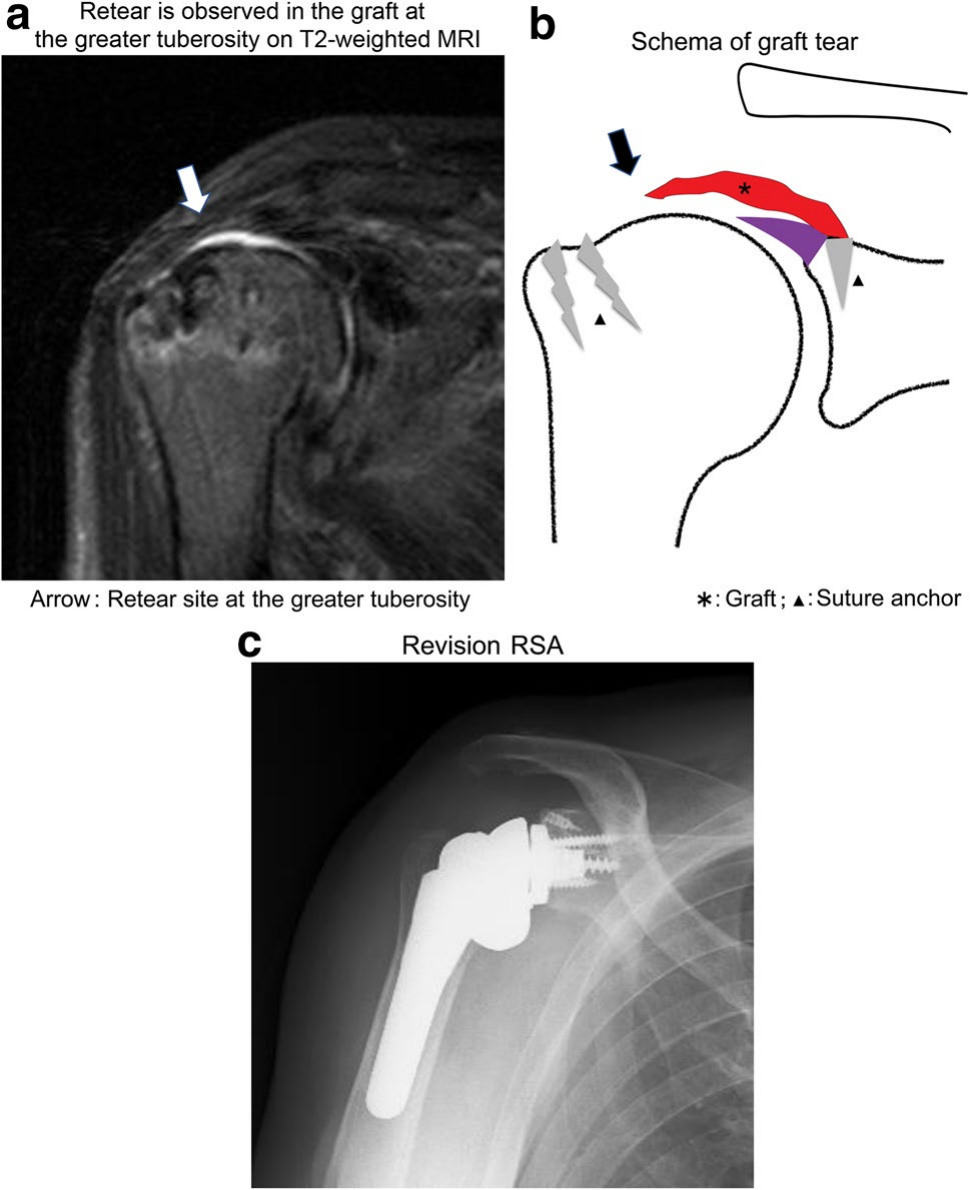
Retear cases. **a** Retear (Sugaya classification type IV) is observed in the graft at the greater tuberosity on T2-weighted MRI. **b** Schema of graft tear. **c** Reverse shoulder arthroplasty was performed 1 year postoperatively

An 81-year-old male underwent SCR for traumatic massive rotator cuff tear caused by a fall. However, MRI performed at 3 months postoperatively revealed retear at the greater tuberosity (Fig. 2a, b). Rehabilitation was continued but no improvement was seen and reverse shoulder arthroplasty was performed 1 year after SCR (Fig. 2c). One year later, the elevation had improved to 120°.

## Discussion

At our hospital, we have been performing arthroscopic patch grafting since 2009 for patients in whom primary repair was impossible. However, the retear rate was 50%. Even if patch grafting is performed on the degenerated remnant tendon, biological repair is difficult. Mori et al. [26] classified cases with Goutallier’s classification ≤ stage 2 (stage 1 or 2) indicating muscle fatty degeneration of the shoulder joint rotator cuff muscles as low grade and those with ≥ stage 3 or 4 as high grade; for low grade cases, the retear rate was reportedly 20.8% in the performed patch surgery. In our case performed in 2016 [16], the retear rate was as high as 50% because 7 of 8 cases of patch surgery were classified as ≥ stage 3. With regard to the degree of retraction of the torn tendon, Boileau et al. [27] classified the stumps of the supraspinatus tendon tears observed under arthroscopy on the coronal plane (stage I, minor retraction located outside the articular cartilage; stage II, moderate head extraction of the humeral head but not reaching the joint cavity; stage III, severe retraction reaching the joint cavity; stage IV, massive tear drawn inside the joint cavity. The stage IV cases in which the degeneration of supraspinatus and infraspinatus was advanced and the stump of the tendon was pulled inward from the glenoid cavity were all re-ruptured in our study), and it was considered as a difficult patch surgery case [15, 16]. Therefore, we switched the surgical strategy to SCR, the procedure developed by Mihata et al. [20] In SCR, superior migration of the humeral head is suppressed and glenohumeral joint stabilization is achieved by attaching the graft to the glenoid superior tubercle and greater tubercle of the humerus [17, 20]. As for the significance of AHD reduction, as suggested by Goutallier et al. [28], AHD suggests humeral head elevation due to rotator cuff tearing and particularly leads to decreased force coupling owing to infraspinatus tendon tearing.

In our case [16], the patch graft showed no improvement in AHD both preoperatively and postoperatively, but SCR did. Audenaert et al. [29] reported that conventional patch grafts that attach the graft with the medial torn tendon stump and the lateral part with greater tuberosity did not show improvement in AHD and that the prevention of humeral head elevation seems difficult using patch grafts. In contrast, in SCR, Mihata et al. [5] and Lim et al. [24] reported that AHD was significantly improved, and this study (Table 2) also showed improvement in AHD both preoperatively and postoperatively. However, in the retear group, no change in AHD was observed, indicating that the humeral head may be elevated, thereby leading to a decrease in clinical outcomes. In SCR, it is important to prevent graft tear.

According to Mihata et al. 2013 [20], the rate of retears was 13% in 24 patients and 23 and 1 patient had Hamada stages 1–3 and stage 4b tears, respectively. The patients with stage 4b tears showed no retear and their acromio–humeral distance improved from 2.4 to 7.2 mm. In our hospital, graft retear rate was 20% (7 of 35 cases); however, of the 35 cases, 9 cases (24%) were either Hamada class 4a or 4b. On including only the cases of Hamada classifications 1–3, retear rate was 7.7% (2 cases). In addition, in our study, neither SSP, ISP nor SubS showed any statistically significant progress or improvement in fatty degeneration preoperatively and postoperatively (Table 2) and these findings were similar to those reported by Mihata et al. [20] and Lim et al. [24]

Moreover, in the grading of preoperative Goutallier scores, no significant differences were observed between the retear and no-retear groups (Table 4). When fatty degeneration is observed on MRI, muscle flexibility and cuff mobility are often reduced preoperatively. It is believed to affect the patients’ choice and postoperative outcomes [30, 31]. At this time, even if equivalent fatty degeneration was observed during SCR, graft survival was not affected.

Mihata et al. reported that 1 out of the 24 cases experienced graft tear [20]; however, no description about the retear site is available.

Lim et al. [24] reported that of 9 graft tear cases, 2 were type 1 (22%) and 7 were type 2 (78%). On the other hand, in our study, of 7 cases, 5 (71.4%) were of type 1 and 2 (28.6%) were of type 2; these findings were contradictory to those of Lim et al. Nakamizo et al. [32] also reported retear in 3 cases (30%) of 10 ASCR (arthroscopic superior capsular reconstruction); they demonstrated that the site where retear occurred had greater tuberosity. As for graft, according to the method used, the thickness and size of the original method of Mihata et al. [18, 33], the method of suturing at the greater tuberosity site, suturing strength, rest fixation period of suturing part, the impact on retear, such as rehabilitation, will be for further study. However, even if these problems are resolved, it is agreeable that patients with advanced arthrosis (Hamada stages 4a and b) are at high risk for retear. At our hospital, multiple regression analyses revealed significant differences in the risk factors for retears; the risk factors, in descending order of importance are as follows: age ≥ 80 years, Hamada stage ≥ 4 and male sex. In elderly individuals, the quality of the graft can be a problem; thus, reconstruction using methods other than grafting might provide better outcomes. Further, via a comparative study of 10 cases of SCR (total 6 cases, including 4 women) and 9 cases of patch grafts (7 of men and 2 of women), Nakamizo et al. [32] reported that 3 cases each (all men) experienced retear. Although this has not been discussed, even this study has become a risk factor for men. Although this aspect has not been examined currently, it seems to be a future research prospect; for example, future studies can assess the differences in the weights of upper limbs between men and women and the differences in the degree of rest after surgery. These risk factors highlight the necessity of carefully selecting the surgical techniques and the patients to be assessed when performing SCR.

Our study has some limitations. First, this was a retrospective study in which SCR was not compared in parallel with conventional patch grafting. Second, because the sample size was small, the number of parameters assessed in the multiple regression analyses was limited. Third, patient and radiographic evaluation may be biased because it has been conducted by three doctors in our hospital. Fourth, both groups (retear and no retear) were not comparable because different preoperative parameters were considered. Finally, only cases with a short-term follow-up period (< 2 years) were included; therefore, only few reports in which the period after development is short could be evaluated in this study. In the future, we hope to report the mid-to-long-term progress of SCR in a larger sample of patients.

## Conclusion

While the postoperative outcomes of superior capsule reconstruction for massive rotator cuff tears were favourable, Furthermore, the SCR was not affected by the residual degeneration of the rotator cuff; it was found that the AHD and the elevation angle were improved if there was no retear of the graft against the massive rotator cuff tear, which was difficult for primary repair. The risk factors for retear after SCR, in descending order are age ≥ 80 years, Hamada stage ≥ 4 and male sex.
